# To grow or not to grow under nutrient scarcity: Target of rapamycin-ethylene is the question

**DOI:** 10.3389/fpls.2022.968665

**Published:** 2022-08-12

**Authors:** María José García, Macarena Angulo, Carlos Lucena, Rafael Pérez-Vicente, Francisco Javier Romera

**Affiliations:** ^1^Department of Agronomy, (DAUCO-María de Maeztu Unit of Excellence), Campus de Excelencia Internacional Agroalimentario, Universidad de Córdoba, Córdoba, Spain; ^2^Department of Botany, Ecology and Plant Physiology, Campus de Excelencia Internacional Agroalimentario, Universidad de Córdoba, Córdoba, Spain

**Keywords:** ethylene, nutrient deficiency responses, nutrient scarcity, plant growth, target of rapamycin (TOR)

## Abstract

To cope with nutrient scarcity, plants generally follow two main complementary strategies. On the one hand, they can slow down growing, mainly shoot growth, to diminish the demand of nutrients. We can call this strategy as “stop growing.” On the other hand, plants can develop different physiological and morphological responses, mainly in their roots, aimed to facilitate the acquisition of nutrients. We can call this second strategy as “searching for nutrients.” Both strategies are compatible and can function simultaneously but the interconnection between them is not yet well-known. In relation to the “stop growing” strategy, it is known that the TOR (Target Of Rapamycin) system is a central regulator of growth in response to nutrients in eukaryotic cells. TOR is a protein complex with kinase activity that promotes protein synthesis and growth while some SnRK (Sucrose non-fermenting 1-Related protein Kinases) and GCN (General Control Non-derepressible) kinases act antagonistically. It is also known that some SnRKs and GCNs are activated by nutrient deficiencies while TOR is active under nutrient sufficiency. In relation to the “searching for nutrients” strategy, it is known that the plant hormone ethylene participates in the activation of many nutrient deficiency responses. In this Mini Review, we discuss the possible role of ethylene as the hub connecting the “stop growing” strategy and the “searching for nutrients” strategy since very recent results also suggest a clear relationship of ethylene with the TOR system.

## Introduction

Terrestrial plants are sessile organisms that have to continuously change their physiology and morphology to adapt to the availability of different nutrients in the soil. To cope with nutrient deficiencies, they can adopt several strategies (Romera et al., 2021), like to stop growth (“stop growing” strategy), to limit the demand of nutrients (Marschner et al., 1996; Shin, 2011; Marzec and Melzer, 2018); to develop physiological and morphological responses (“searching for nutrients” strategy), to facilitate the acquisition of the deficient nutrient(s) (Kobayashi and Nishizawa, 2012; Zhang et al., 2014; García et al., 2015; Romera et al., 2021); to recycle the deficient nutrient(s) from the oldest leaves and organs to the youngest ones (Masclaux-Daubresse et al., 2017; Chen et al., 2019); and to substitute the deficient nutrient(s) by other(s) that can play a similar role (Wakeel et al., 2011; Lambers et al., 2012; Waters et al., 2012). These different strategies are not incompatible between them and can occur simultaneously. This Mini Review will focus on the two first strategies, the “stop growing” strategy and the “searching for nutrients” strategy, whose interrelationship is not yet known. It is clear that, if plants dedicate resources to activate nutrient deficiency responses, they must take away them from other functions, such as growth. In fact, iron (Fe) efficient soybean varieties offer lower yields than the inefficient ones when grown under Fe sufficient conditions (Atwood et al., 2014). The “stop growing” strategy is frequently used by wild plant genotypes adapted to impoverished soils since, in this way, they can limit the demand of nutrients without showing any symptoms of deficiency. In crop plants, however, the “stop growing” strategy can have undesirable side effects because their yield may be compromised. On the other hand, if crop plant genotypes keep growing despite nutrient deficiencies, this can lead to clear symptoms of deficiency. Thus, it is necessary a balance between both kind of strategies, in such a way that crop plants can acquire the nutrients they need by means of the “searching for nutrients” strategy without compromising their growth and yield by the “stop growing” strategy.

**FIGURE 1:**
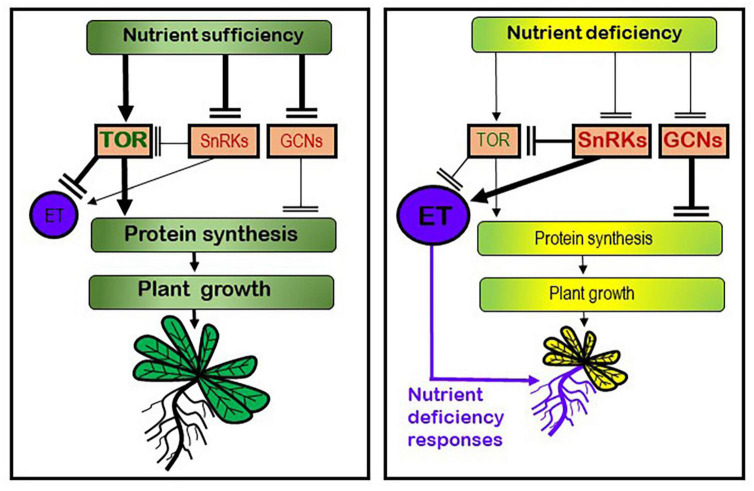
Working model to explain the possible roles of the TOR/SnRKs/GCNs regulatory system and ethylene (ET) in plant growth and development of nutrient deficiency responses. Under nutrient sufficiency, the TOR kinase is activated leading to an increase of protein synthesis and meristem activity, and consequently to a promotion of plant growth. In this condition, TOR blocks ET synthesis and signaling. Under nutrient deficiency, other kinases, like SnRKs and GCNs, are activated leading to inhibition of TOR, protein synthesis, meristem activity, and growth. In this condition, ET synthesis and signaling is enhanced, which leads to the activation of nutrient deficiency responses, mainly in roots, aimed at acquiring the deficient nutrient from the medium. Based on Robaglia et al. (2012), García et al. (2015, 2021a,b), Lucena et al. (2015), Dobrenel et al. (2016a), Rodríguez et al. (2019), Zhuo et al. (2020), Fu et al. (2021), and Romera et al. (2021).

In yeasts and animals, growth is to a large degree controlled by the TOR system, which is a protein complex with kinase activity that stimulates protein synthesis and growth when there are enough nutrients and other favorable conditions. In plants, although less studied, the TOR pathway integrates hormonal and nutritional signals to regulate plant growth and development (Robaglia et al., 2012; Barrada et al., 2015; Dobrenel et al., 2016a; Rosenberger and Chen, 2018; Ryabova et al., 2019; Wu et al., 2019; Fu et al., 2020). In relation to the “searching for nutrients” strategy, it is known that plants can develop morphological and physiological responses, mainly in their roots, aimed to facilitate mobilization and acquisition of the deficient nutrient(s) (Zhang et al., 2014; García et al., 2015, 2021a,b; Romera et al., 2021). These responses are activated when plants suffer from a nutrient deficiency and are switched off once the nutrient has been acquired in sufficient quantity. Several hormones and signaling molecules, like ethylene (ET), auxin and nitric oxide (NO), have been implicated as activators of most of them (Romera and Alcántara, 2004; Jung et al., 2009; García et al., 2011, 2015; Lucena et al., 2015; Romera et al., 2016, 2017). Very recently, it has been found that TOR can affect ET synthesis and signaling (Dong et al., 2015; Zhuo et al., 2020; Fu et al., 2021), which indicates that both strategies (“stop growing” and “searching for nutrients”) could be interconnected through TOR and ET (Figure 1).

## Nutrient deficiencies and the “stop growing” strategy

As a general rule, the growth of plants is related to the quantity of nutrients they receive, in such a way that growth linearly increases when a determined nutrient augments until reaching an optimum and then decreases because of toxicity problems (Marschner, 2012). When nutrients are deficient, a frequent adaptive response of plants is the inhibition of shoot growth, to limit the demand of nutrients (Marschner et al., 1996; Shin, 2011). Root growth is often less inhibited, which increases the root/shoot ratio and consequently the capacity of roots to fulfill the demand of the deficient nutrient (Marschner et al., 1996; Borch et al., 1999; Shin, 2011; Marzec and Melzer, 2018).

Nutritional disorders that reduce growth and yield are generally characterized by specific visible symptoms. However, in some cases plants do not show apparent symptoms upon a nutrient deficiency because they stop growing and suffer a latent deficiency. The effects of the different nutrients on growth are not exactly the same. Some nutrient deficiencies affect more the youngest leaves and the apical meristems, such as calcium (Ca), boron (B), or iron (Fe) deficiency, while other ones affect more the oldest leaves, such as nitrogen (N), phosphorus (P), or potassium (K) deficiency (Marschner, 2012). Similarly, root growth is differently affected depending on the nutrient deficiencies (Gruber et al., 2013; Singh et al., 2018; Bouain et al., 2019).

## Nutrient deficiencies and the “searching for nutrients” strategy

As previously stated, to cope with nutrient deficiencies plants can develop physiological and morphological responses, mainly in roots, aimed to facilitate the acquisition of the deficient nutrient(s) (Shin, 2011; Kobayashi and Nishizawa, 2012; Zhang et al., 2014; García et al., 2015, 2021a,b; Romera et al., 2021). Following are some of the most frequent physiological and morphological responses.

### Physiological responses

Physiological responses include acidification of the rhizosphere; increased expression of specific nutrient transporters, both at the root epidermis and in other tissues; increased synthesis and/or release of chelating agents into the medium (to improve mobilization of nutrients in the soil and/or inside the plant); increased expression of transporters associated with the release of different compounds to the medium; enhancement of some root enzymatic activities, like ferric reductase activity and phosphatase activity; and others (Zhang et al., 2014; García et al., 2015, 2021a,b; Lucena et al., 2015, 2019; Romera et al., 2021).

### Morphological responses

Morphological responses include modifications of the root system architecture, related to primary root elongation and development of lateral roots; development of root hairs; of cluster roots (also named proteoid roots); and of root transfer cells. Most of these root modifications enhance nutrient uptake by increasing the surface of contact of roots with soil (Wang et al., 2014; García et al., 2015). In many cases, both physiological and morphological responses are located in the subapical regions of the roots where they act synergistically (Romera and Alcántara, 2004; Jung et al., 2009; García et al., 2011; Wang et al., 2014; Lucena et al., 2015).

### Ethylene involvement in the regulation of physiological and morphological responses

Once the deficient nutrient has been acquired in sufficient quantity, the responses need to be switched off to avoid nutrient toxicity and energy costs. Their regulation is not totally known but several hormones and signaling molecules, such as ET, auxin, cytokinins, and NO, have been involved in their control (Krouk et al., 2011; Iqbal et al., 2013; García et al., 2015; Romera et al., 2017, 2021). ET has been involved in the activation of both physiological and morphological responses, like the acidification response, the expression of nutrient transporters, the release of phenolic compounds and the development of root hairs, to several nutrient deficiencies, such as P, Fe, K, Mg, and B (you can find abundant information about this in the following reviews and in the references therein: Romera and Alcántara, 2004; García et al., 2015; Lucena et al., 2015; Romera et al., 2016, 2017, 2021; Li and Lan, 2017).

Ethylene is synthesized from methionine *via* a pathway where ACC synthases (encoded by *ACS* genes) and ACC oxidases (encoded by *ACO* genes) participate, and where 1-aminocyclopropane-1-carboxylic acid (ACC) is the immediate ET precursor (Sauter et al., 2013). Although ET’s mode of action is not fully understood, a linear canonical signaling pathway has been proposed in Arabidopsis (Binder, 2020):

ET—||ET receptors → CTR1—||EIN2 → EIN3/EILs → ERFs → ET responses

**FIGURE 2:**
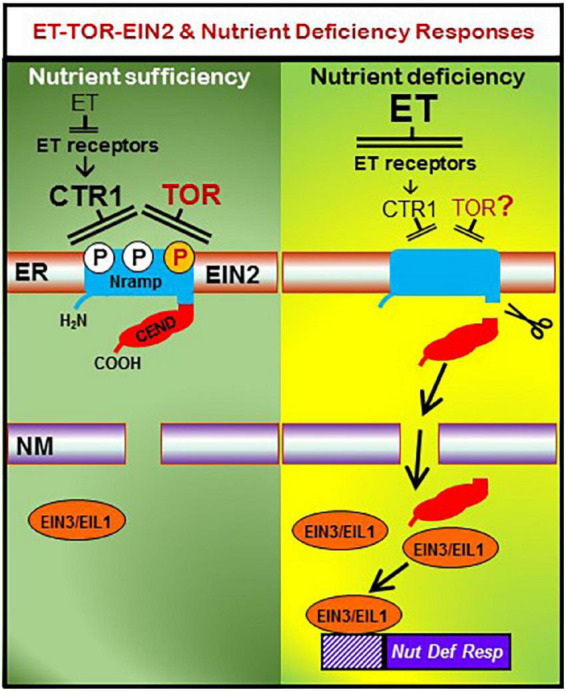
Working model to explain the possible roles of ethylene (ET) and TOR in the activation of nutrient deficiency responses through EIN2. Left: in the absence of ethylene (ET), which occurs under nutrient sufficiency, the CTR1 kinase phosphorylates EIN2 at Ser645/Ser924, preventing the cleavage and translocation of CEND into the nucleus. In parallel, under nutrient sufficiency, the TOR kinase phosphorylates EIN2 at Thr657, preventing the cleavage and translocation of CEND into the nucleus. Right: under nutrient deficiency, ET production is enhanced and, once perceived by ET receptors, CTR1 is inactivated, thus resulting in dephosphorylation of EIN2 and its cleavage. CEND is then translocated into the nucleus, where results in more EIN3/EIL1 binding to target genes and ultimately transcription activation of ET responsive genes, like those related to nutrient deficiency responses. Similarly, under nutrient deficiency, TOR is inactivated, which could also result in dephosphorylation of EIN2 and its cleavage and translocation into the nucleus. Whether the influence of TOR on EIN2 plays a key role in the activation or deactivation of nutrient deficiency responses deserves further research. ER, Endoplasmic Reticulum; NM, Nuclear Membrane. Based on Angulo et al. (2021) and Fu et al. (2021).

In this pathway, CTR1 is a kinase, EIN2 is a protein located in the endoplasmic reticulum (ER) membrane, and EIN3, EILs and ERFs are transcription factors (Binder, 2020). EIN2 possesses a Nramp-like transmembrane domain and a cytosolic COOH end (CEND; Figure 2) domain (Binder, 2020). In the absence of ET, CTR1 phosphorylates EIN2, preventing the cleavage and translocation of CEND into the nucleus. In the presence of ET, CTR1 is inactivated, resulting in dephosphorylation of EIN2 and its cleavage. CEND is then translocated into the nucleus, where it finally enhances EIN3/EIL1 binding to target genes and transcription activation (Figure 2; Binder, 2020; Angulo et al., 2021).

## Target of rapamycin and the “stop growing” strategy

The general control of plant growth has been associated with some protein kinases, like Target Of Rapamycin (TOR), Sucrose non-fermenting 1-Related protein Kinases (SnRK1, SnRK2) and General Control Non-derepressible (GCN) kinases (GCN1, GCN2, GCN20; Robaglia et al., 2012; Barrada et al., 2015; Dobrenel et al., 2016a; Sesma et al., 2017; Rosenberger and Chen, 2018; Shi et al., 2018; Ryabova et al., 2019; Li et al., 2021). Under nutrient sufficiency and other favorable conditions, TOR promotes growth by activating meristems and anabolic processes, such as protein synthesis, and by repressing catabolic processes, such as autophagy (Figure 1; Barrada et al., 2015; Dobrenel et al., 2016a; Sesma et al., 2017; Rosenberger and Chen, 2018; Shi et al., 2018; Chen et al., 2019; Rodríguez et al., 2019; Ryabova et al., 2019; Couso et al., 2020). It should be noted that protein synthesis is one of the most energetically requiring processes (Dong et al., 2017; Romero et al., 2020). In contrast, nutrient deficiency activates other kinases, like SnRKs and GCNs, that act antagonistically to TOR, thus repressing protein synthesis and growth (Figure 1; Robaglia et al., 2012; Barrada et al., 2015; Dobrenel et al., 2016a,b; Wang et al., 2017; Rosenberger and Chen, 2018; Shi et al., 2018; Chen et al., 2019; Rodríguez et al., 2019; Ryabova et al., 2019; Li et al., 2021). The ways TOR, SnRKs, and GCNs regulate protein synthesis is related to their capacity to control processes like ribosome biogenesis and mRNA translation (Echevarría-Zomeño et al., 2013; Dobrenel et al., 2016b; Dong et al., 2017; Sesma et al., 2017; Caballero-Molada et al., 2018; Faus et al., 2018; Shi et al., 2018; Rodríguez et al., 2019; Ryabova et al., 2019; Couso et al., 2020; Romero et al., 2020). For example, GCN2 phosphorylates the eIF2 factor, necessary to initiate mRNA translation, preventing it (Lageix et al., 2008; Sesma et al., 2017; Caballero-Molada et al., 2018; Faus et al., 2018; Romero et al., 2020). In plants, SnRKs can interact and phosphorylate RAPTOR, inhibiting TOR activity (Figure 1; Rodríguez et al., 2019 and references therein; Li et al., 2021 and references therein). However, the direct interaction between TOR and GCN2 is not yet clear in plants (Lageix et al., 2008).

In yeast and mammals, the TOR system comprises two protein complexes (TORC1 and TORC2) while in plants is only present the TORC1 complex, composed of TOR, RAPTOR (“Regulatory Associated Protein of TOR”) and LST8 (“Lethal with Sec Thirteen8”; Robaglia et al., 2012; Salem et al., 2018; Shi et al., 2018; Zhang et al., 2018; Rodríguez et al., 2019; Ryabova et al., 2019; Salem and Giavalisco, 2019). In most plant species, including Arabidopsis, TOR is encoded by a single gene (Menand et al., 2002; Dobrenel et al., 2016a). However, in Arabidopsis there are two RAPTOR genes, RAPTOR1A and RAPTOR1B, and two LST8 genes, LST8-1 and LST8-2, being RAPTOR1B (also named RAPTOR3G) and LST8-1 the most expressed ones (Moreau et al., 2012; Zhang et al., 2018; McCready et al., 2020; Pereyra et al., 2020). In plants, the TOR gene is not expressed in all tissues but in shoot and root meristems where plays a key role in their activity (Menand et al., 2002; Xiong et al., 2013; Li et al., 2017; Ryabova et al., 2019).

## Target of rapamycin and the “searching for nutrients” strategy

The relationship between the TOR system and the responses to nutrient deficiencies has not been deeply studied. However, there are several experimental results suggesting an interconnection between them, both in algae and in higher plants. In *Chlamydomonas reinhardtii* it has been found that the TORC1 complex activity depends on P availability (Couso et al., 2020). In Arabidopsis, TOR inhibition affects the expression of Fe-related genes as well as the development of root hairs, a typical morphological response to Fe deficiency (Ren et al., 2012). Besides Fe and P, TOR also affects K, S, and N assimilation (Xiong et al., 2013; Salem et al., 2018; Shi et al., 2018; Wu et al., 2019; Fu et al., 2020; Ingargiola et al., 2020). TOR signaling activates transcription of genes involved in sulfur assimilation and transport, like the sulfate transporter *SULTR1.2*, as well as genes involved in nitrogen assimilation and transport, like the nitrate transporter *NRT1.1* (Xiong et al., 2013; Fu et al., 2020). By contrast, some nutrient deficiencies, like sulfur deficiency, affect TOR activity, probably through glucose energy signaling (Dong et al., 2017). Similarly, TOR is inhibited in nitrogen-deprived seedlings, and quickly reactivated upon resupply of either nitrate, ammonium, or amino acids (Liu et al., 2021).

Besides TOR, SnRKs and GCNs have also been related to nutrient stresses. In relation to SnRKs, it has been found that SnRK2.1 plays a key role in the signaling cascade leading to S deprivation responses in *Chlamydomonas* (Gonzalez-Ballester et al., 2008). In the same way, the *Arabidopsis* AtSnRK2.2, AtSnRK2.3, and AtSnRK2.4, and the *Triticum polonicum* TpSnRK2.10 and TpSnRK2.11 have been linked to the expression of several metal-related genes under Cd stress (Kulik et al., 2012; Fan et al., 2014; Wang et al., 2019). In relation to GCNs, there are several results suggesting a role for them on Fe nutrition, most of them in yeast (Caballero-Molada et al., 2018; Faus et al., 2018; Romero et al., 2020). In dicot plants, such as *Glycine max* and *Medicago truncatula*, it has also been found a relationship between GCN2 and Fe deficiency, since *GCN2* expression is enhanced in both roots and leaves of Fe-deficient plants (Santos et al., 2016). GCN2 activation usually requires the participation of other GCN proteins, like GCN1 and GCN20 (Wang et al., 2017; Faus et al., 2018; Romero et al., 2020). In Arabidopsis, the GCN1 protein is also named ILITHYIA (ILA), and the *gcn1* (*ila*) mutants present chlorosis of youngest leaves and shorter roots, both symptoms related to Fe deficiency (Faus et al., 2018).

## Relationship between target of rapamycin and ethylene

As previously stated, ET is a key player in the activation of physiological and morphological responses to several nutrient deficiencies. Moreover, EIN2 and EIN3, two components of the ET transduction pathway, play crucial roles in such an activation (Angulo et al., 2021; García et al., 2021a,b). Consequently, ET is a critical signal for the regulation of nutrient deficiency responses. In addition, ET could have a very close relationship with the TOR/SnRKs/GCNs system: TOR inhibition positively affects ET (Figure 1) by up-regulating ET-synthesis genes, like *ACS* and *ACO* genes, and ET-signaling genes, like *ERF* genes (Dong et al., 2015; Zhuo et al., 2020); SnRK1 affects ET synthesis and signaling (Figure 1; Rodríguez et al., 2019 and references therein; Jamsheer et al., 2021 and references therein); and ACC (ET precursor) up-regulates *GCN2* expression in Arabidopsis (Lageix et al., 2008). In general, when TOR activity is inhibited, and consequently growth, genes related to hormones that promote growth, like auxin and brassinosteroids, are repressed, while those that inhibit growth, like ET and abscisic acid (ABA), are up-regulated (Dong et al., 2015; Burkart and Brandizzi, 2021).

Very recently, a close link between TOR and ET has been reported. Fu et al. (2021) have found that EIN2, a critical component of the ET transduction pathway is a target of TOR. TOR is a kinase that could act in parallel to the CTR1 kinase, belonging to the canonical ET signaling pathway (Figure 2; Binder, 2020; Fu et al., 2021). As previously described, in the absence of ET, CTR1 phosphorylates EIN2, preventing the cleavage and translocation of its CEND portion into the nucleus. Similarly, TOR can phosphorylate EIN2 (at other phosphorylation sites than CTR1) and thereby could affect the cleavage and translocation of the CEND portion into the nucleus thus affecting processes downstream EIN2 (Figure 2; Fu et al., 2021). These results and the ones described in the above paragraph suggest a clear interrelationship between ET and the TOR system.

In conclusion, ET can be envisioned as a hub connecting the “searching for nutrients” strategy, since it is a key player in the activation of many nutrient deficiency responses, and the “stop growing” strategy, since its synthesis and signaling is negatively regulated by TOR. Moreover, ET itself can be an inhibitor of growth (Street et al., 2015).

## Concluding remarks and future perspectives

The interrelation between the “stop growing” and the “searching for nutrients” strategy is a very important question for crop plants since, under nutrient deficiencies, they should keep growing to avoid a drastic reduction of their yield. However, this can only be done if they have the ability to develop efficient nutrient deficiency responses. TOR is implicated in the control of plant growth, and ET in the activation of nutrient deficiency responses. Since TOR can negatively affect ET synthesis and signaling, it is tempting to speculate that both strategies (“stop growing” and “searching for nutrients”) should be interrelated through the interdependency of TOR and ET, as suggested in Figures 1, 2. Nonetheless, this interaction has been barely studied and needs further research to answer questions like the following ones: Does the modification of TOR affect the activation of nutrient deficiency responses through EIN2 (Figure 2)? Does the onset of nutrient deficiency responses affect TOR? Do nutrient deficiencies affect TOR in the same way at the early stages of the deficiency and later on? Obviously, the working models depicted in Figures 1, 2 are simplified models because hormones and factors others than TOR and ET can influence growth and nutrient deficiency responses. The understanding of the interrelation between TOR and ET in the control of plant growth and development of nutrient deficiency responses is complex and has just begun.

## Author’s note

We apologize to authors whose works were not cited in this review due to manuscript length restrictions.

## Author contributions

MG and FR wrote a first draft of the manuscript that was corrected and improved by all the authors. All authors revised the information related to TOR, ET, and nutrient deficiency responses and approved the submitted version.
